# Electric-Scooter- and Bicycle-Related Trauma in a Hungarian Level-1 Trauma Center—A Retrospective 1-Year Study

**DOI:** 10.3390/jcm14248782

**Published:** 2025-12-11

**Authors:** Viktor Foglar, Dávid Süvegh, Mohammad Walid Al-Smadi, Daniel Veres, Csenge Nemes, Árpád Viola

**Affiliations:** 1 Department of Neurosurgery and Neurotraumatology, Dr. Manninger Jenő Traumatology Institution, 1081 Budapest, Hungaryarpadviola@gmail.com (Á.V.); 2 Department of Neurotraumatology, Semmelweis University, 1081 Budapest, Hungary; 3 Department of Biophysics and Radiation Biology, Semmelweis University, 1094 Budapest, Hungary

**Keywords:** electric scooter, emergency care, trauma care, injury, scooter, bicycle, alcohol consumption, traffic crash, injury patterns, craniofacial injury

## Abstract

**Background/Objectives:** In recent years, electric scooters have gained widespread popularity as an easy and affordable mode of transport in urban areas worldwide. Simultaneously, trauma centers have observed an increasing number of associated injuries to users. While injury patterns associated with other vehicles are now well-researched, electric-scooter-related injuries are a new topic in the literature. Our study aims to investigate the differences in injury patterns and other critical crash characteristics among riders of bicycles, electric scooters, and scooters. **Methods:** This one-year retrospective observational study examined patients who sustained injuries while riding bicycles, electric scooters, or scooters between April 2021 and March 2022 at Hungary’s largest trauma center in Budapest. During this one-year period, we identified 1938 patients, 1378 cyclists, 370 electric scooter users, and 190 scooter users. Basic demographic information, recorded injury type and severity, time of day the injury occurred, and alcohol usage were recorded as outcome measures. **Results:** While 4.6% of cyclists and 5.8% of scooter riders had consumed alcohol, 26.8% of electric scooter riders were under the influence of alcohol at the time of their crash. Of electric-scooter-related injuries, 45.8% occurred at night, compared to only 9.2% and 14.1% of bike and scooter-related injuries, respectively. E-scooter crashes constituted 19.1% of total cases but surged to 52.3% at night. Patients under the influence of alcohol were much more likely to experience mild head injuries (*p* < 0.0001) and severe head injuries (*p* < 0.0001), but less likely to suffer mild limb injuries (*p* < 0.0001) and severe limb injuries (*p* < 0.0001) compared with sober patients. Cyclists had significantly 3 times fewer cases of severe head trauma than those injured while using electric scooters (*p* = 0.0166). **Conclusions:** The study highlights a significant risk of severe craniofacial injuries in e-scooter users after consuming alcohol, exceeding that in sober riders and cyclists. Predominantly occurring at night, these injuries are closely linked with alcohol use. The findings advocate for mandatory helmet laws and stricter regulations on e-scooter use to enhance safety, especially at night.

## 1. Introduction

The rapid urbanization of cities worldwide has brought advancements in transportation and unprecedented challenges. Micro-mobility devices (public-sharing electric scooters (e-scooters) and electric bikes) have become increasingly popular worldwide, offering a convenient and affordable solution to this “first/last mile” problem while also reducing the carbon footprint of cities [1,2]. Following their initial widespread introduction in the United States (US) in 2017, e-scooters experienced a surge, with reports indicating over 38.5 million units in 2018 and a subsequent rise in European adoption [3,4].

However, these benefits are accompanied by substantial risks. Recent studies have highlighted a significant increase in e-scooter-related injuries [5,6,7,8]. Crashes are often associated with high speeds (30–35 km/h) and alcohol consumption, which impairs reaction time and balance [9,10,11]. Riders are subject to severe injuries such as traumatic brain injuries and craniomaxillofacial and limb fractures [12,13]. The introduction of shared e-scooter services has been linked to an increase in overall traffic accidents, although cities with more extensive cycling infrastructure experience smaller increases, highlighting the role of urban planning in reducing injury risk [14]. Beyond epidemiological trends, vehicle biomechanics also raise injury risks. E-scooters’ small wheels heighten instability on uneven surfaces, increasing the likelihood of falls, while larger wheels enhance safety and comfort [15].

Rising injuries, influenced by both increasing usage and vehicle design, present growing public health and trauma care challenges. In the U.S., e-scooter injuries increased by 222% between 2014 and 2018, with hospital admissions rising 365% [16], highlighting both health and economic impacts. Beyond public health concerns, the costs are significant—New Zealand reported 1.3 million NZD in e-scooter injuries over seven months, averaging 1300 NZD per scooter [17,18,19].

E-scooters were introduced in Hungary in 2019, yet the absence of specific regulations has hindered proper classification and accurate crash data collection [20]. Current regulations neither mandate helmet usage nor prohibit the operation of e-scooters while intoxicated. Due to the absence of a distinct legal category for e-scooters, they are often treated similarly to bicycles. This legislative ambiguity is not unique to Hungary; cities like Paris have acted decisively by choosing to ban these vehicles from September 2023 [21], highlighting the pressing need to address e-scooter safety [12].

Similar to e-scooters, electric bikes pose a high risk of injury to their users, as the increasing number of accidents has become a public health issue in many countries. Studies show that users can suffer high-energy maxillofacial, spinal, and orthopedic trauma, therefore advocating for stricter helmet and protective equipment usage [22]. Since electric bikes, especially rental electric bikes, are particularly rare in Hungary, this study does not delve into the topic in greater detail.

Focusing on electric scooter injuries, it is important to note the lack of previous studies directly comparing injuries from bicycles, scooters, and electric scooters over an identical one-year timeframe. This gap highlights the novelty of our study, which examines differing risk factors and injury severities, aiming to improve understanding of urban transport-related injuries and inform targeted safety measures and policymaking.

This study aims to analyze the injury patterns and crash characteristics of electric scooters (e-scooters), scooters, and bicycles in a Hungarian level-1 trauma center, the Dr. Manninger Jenő Trauma Center, over a one-year period. Our goal is to understand the unique and shared injury profiles among these modes of transport to inform safer urban transportation policies and trauma care protocols in Hungary.

## 2. Materials and Methods

This is a retrospective observational study conducted on 1938 patients who presented to Hungary’s largest trauma center between 1 April 2021, and 31 March 2022, with injuries from riding a bicycle, e-scooter, or scooter. All patients with incomplete medical documentation (mainly due to discharge against medical advice) were excluded from the study. A database was created, categorizing patients by their vehicle type: bicycle, standing electric scooter (e-scooter), and non-motorized scooter (scooter). From medical documentation, age, sex, injury date, presentation time, alcohol influence, hospitalization need, duration, and injury type and severity were recorded. Data on alcohol consumption were self-reported, as it is not common practice to measure blood alcohol concentration (BAC) for trauma patients in our hospital unless it is directly necessary for patient management and care. In this study, alcohol use was treated as an exposure variable—examined for its association with injury severity and type—rather than as an outcome measure. Presentation times were grouped into four categories: mornings (5 a.m.–10 a.m.), daytime (10 a.m.–6 p.m.), evenings (6 p.m.–10 p.m.), and nighttime (10 p.m.–5 a.m.), with intervals adjusted proportionally to each category. The main outcome measures were the type and severity of the sustained injury, presentation time, alcohol consumption, and need for hospitalization.

Injuries were categorized based on the affected regions: extremities, head and face, spine, and trunk (encompassing the thoracic and abdominal areas). For diagnosis, physical examinations and basic X-ray scans were employed for minor injuries, while computed tomography (CT) scans were used for more severe cases. Injuries were further classified as mild or severe. Mild injuries to limbs included contusions, abrasions, and superficial wounds without deep tissue damage, while severe ones involved fractures or tears in ligaments or muscles. Mild head injuries comprised superficial wounds and contusions in the craniomaxillofacial region, whereas severe ones involved fractures or intracranial bleeds like epidural, subdural, subarachnoid, and parenchymal hemorrhages. Mild core injuries were superficial wounds and contusions, while severe ones included fractures, penetrating wounds, or organ damage. Spinal injuries ranged from distortions and dislocations to fractures. The categories “mild” and “severe” are not exclusive in each region. If, and practically in all cases of a severe injury, mild injury to the affected region is also present, the patient is noted to have “mild” and “severe” trauma to that region. During the statistical analysis of the injury types, we used patients with no trauma to the given region as the comparator group for accurate comparison. Although this study employed a simplified, region-based injury classification reflecting routine clinical workflow, the categorization of mild and severe trauma broadly parallels the Injury Severity Score (ISS) system, which provides a standardized measure of overall injury burden [23,24].

Statistical analysis of the database involved fitting a logistic regression model for each trauma type and severity as outcome variables. Predictors were vehicle type, alcohol consumption, and presentation time, controlling for age and sex. Multicollinearity, interactions, and age non-linearity were assessed, but no relevant ones were found. Results were reported with Wald-type *p*-values, adjusted odds ratios (OR), and 95% confidence intervals (CI). Variable inclusion was based on clinical relevance and previously reported associations with trauma outcomes; age and sex were included to adjust for potential confounding effects. For categorical variables with multiple levels (more than 2), Tukey-adjusted *p*-values and marginal mean differences are reported. All analyses utilized R (R Core Team 2023, v4.2.3), rms (Harrell, Jr. 2023), emmeans (Lenth, 2023), DHARMa (Hartig, 2022), and car (Fox, Weisberg, and Price 2023) packages.

The study was conducted in accordance with the Declaration of Helsinki and approved by the Institutional Review Board of Péterfy Sándor Utcai Kórház-Rendelőintézet és Baleseti Központ (Registration number: 02/2021, 6 January 2021).

## 3. Results

During the one year, we observed and treated 1938 patients for injuries related to various types of micro-mobility devices. Specifically, 1378 (71.1%) patients visited our trauma center after accidents involving bicycles, 370 (19.1%) after accidents involving e-scooters, and 190 (9.8%) with non-motorized scooter-related injuries. The mean age of injured cyclists was 37.9 years (SD: 14.9 years), e-scooter (mean: 31.3 years, SD: 10.1 years), and scooter (mean: 30.6 years, SD: 13.7 years) users. Male predominance was noted among all groups: 69.2% in the bicycle group, 66.2% among e-scooter users, and 58.9% in the scooter group (Table 1).

Regarding the timing of injuries, 8.3% of bicycle injuries happened in the morning, 43.3% in the daytime, 39.2% in the evening, and 9.2% at night-time. The proportions for the e-scooter group were 6.8%, 21.3%, 26.0%, and 45.8%, while for the scooter group, they were 9.2%, 42.0%, 34.6%, and 14.1%, respectively. (Table 1) While the proportion of e-scooter crashes among all cases was 19.1%, during night-time, 52.3% of all crashes happened using e-scooters.

Before their crashes, 4.6% of cyclists consumed alcohol, 26.8% of e-scooter users, and 5.8% of scooter users. The hospital admission rates were 10.3% for the bicycle group, 14.6% for the e-scooter group, and 6.8% for the scooter group. (Table 1) The median hospital stay was 2 days for all three groups.

**Figure 1:**
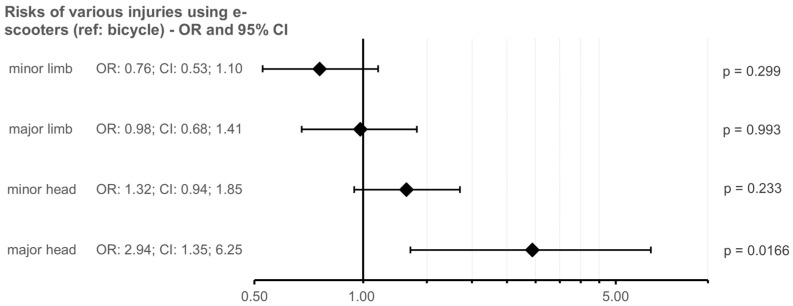
Forest plot illustrating the relative risk of injury types among e-scooter users compared to cyclists (reference group). E-scooter users had a significantly higher risk of major head injuries. OR = odds ratio, CI = confidence interval, ref = reference group.

Regarding injury severity, 78.9% of cyclists suffered mild extremity trauma, and 25.3% had severe extremity trauma. No significant difference was noted between e-scooter (70.8% mild, 25.4% severe) and scooter groups (74.7% mild, 23.7% severe) (Figure 1). However, in the bicycle group, 29.9% suffered mild facial or cranial injuries and 3.3% sustained severe facial or cranial injuries, while the rates were 50.8% and 9.2% in the e-scooter group. Severe head trauma was significantly more common in e-scooter users compared with cyclists (OR: 2.94, CI: 1.35; 6.25, *p* = 0.0166, Figure 1). Nevertheless, 36.8% and 5.8% of the scooter group suffered these types of injuries, with no significant difference from the other groups.

Spinal injuries were present in 4.28% of cyclists, 1.9% of e-scooter users, and 1.1% of scooter users. Mild and severe abdominal or thoracic trauma were reported in 13.4% and 2.1% of cyclists, 9.2% and 1.9% of e-scooter users, and 6.8% and 0.5% of scooter users (Table 2).

For mild limb injuries, 79.8% of sober and 44.7% of patients who consumed alcohol suffered such injuries (OR: 3.85, CI: 2.62; 5.65, *p* < 0.0001). For severe limb injury, these rates were 26.5% and 11.6%, respectively (OR: 3.26, CI: 1.94; 5.47, *p* < 0.0001).

**Figure 2:**
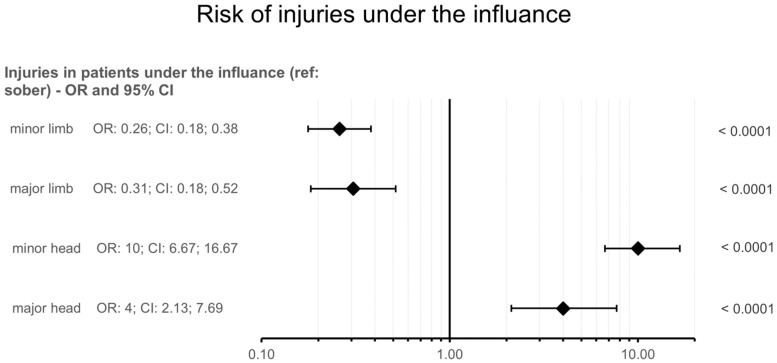
Forest plot showing the risk of different injury types in patients under the influence of alcohol compared with sober patients. OR = odds ratio, CI = confidence interval, ref = reference group.

Patients under the influence of alcohol had a markedly higher risk of head injuries: a 10-fold higher risk of mild head trauma (OR: 10.0, CI: 6.67; 16.7, *p* < 0.0001) and a 4-fold higher risk of severe head trauma (OR: 4.0, CI: 2.13; 7.69, *p* < 0.0001) compared with sober patients. Mild body trauma was experienced by 12.6% of sober patients and 4.6% of drunk patients (OR: 3.2, CI: 1.42; 7.21, *p* = 0.0051). For other injuries, no significant effect of alcohol consumption was found. (Figure 2).

### In-Hospital Mortality, Surgery Rates, and GCS Distributions Across Head Injury Groups

As we assessed the clinical outcomes from all patients with head injury (n = 670), we found that two fatalities (0.299%) occurred, both in individuals with severe and mild head trauma after bicycle accidents. One of them was an 81-year-old male, the other an 82-year-old female; neither of them wore helmets at the time of their accidents. They had GCS 12 and GCS 9 on admission to the hospital, and both underwent emergency surgery. Despite all efforts, the patients died within days due to the severity of their injuries.

In the case of a patient with a mild head injury sustained while riding an e-scooter, the hospital documentation was not available; therefore, the exact outcome is unknown. The remaining patients either underwent surgical intervention or were treated conservatively and were subsequently discharged from the hospital in satisfactory clinical condition. Those who did not require further neurosurgical or trauma care were transferred in stable condition to the regionally designated hospitals for specialty treatment, including oral and maxillofacial surgery, otorhinolaryngology, or ophthalmology.

Among patients with electric-scooter-related head injuries, 34 were classified as severe and 188 as mild. Most severe cases presented with a GCS of 15 (91.17%), while one patient (2.94%) had a GCS of 12. No in-hospital deaths occurred, and the mean GCS was 14.85 ± 0.56. Any surgical intervention was required in 2 patients (5.88%). Among mild cases, 96.81% had a GCS of 15, with no deaths and a mean GCS of 14.94 ± 0.46; surgery was performed in 9 patients (4.79%).

For bicycle-related head injuries, there were 45 severe and 412 mild cases. In the severe group, 95.56% presented with a GCS of 15, and 2 patients (4.44%) died during hospitalization. The mean GCS was 14.80 ± 0.99, and 6 patients (13.33%) underwent surgery. Among mild bicycle-related injuries, nearly all patients (98.79%) had a GCS of 15, with a mean of 14.97 ± 0.35; 2 patients (0.48%) died, and 15 (3.64%) required surgery.

In the scooter-related head injury group, 11 severe and 70 mild cases were identified. In the severe subgroup, 90.91% of patients had a GCS of 15, and one patient (9.09%) had a GCS of 14. No in-hospital deaths or surgeries occurred. The mean GCS was 14.91 ± 0.30. Among mild cases, 95.71% had a GCS of 15, with no fatalities or surgical interventions, and a mean GCS of 14.93 ± 0.39 (Table 3).

## 4. Discussion

Based on our results and those of previous studies, it is evident that e-scooters have resulted in an increase in the number of patients admitted to trauma care centers globally, suffering from distinctive injury patterns, especially head injuries [25]. In Hungary, a similar trend can be observed. This may be partly explained by the fact that, in many places, these devices are used in an intoxicated state due to regulatory deficiencies [1,4,8,11,26,27]. The heightened interest of clinicians in this topic is illustrated by numerous publications on e-rollers over the last few years [25]. While previous studies have mainly focused on injury patterns associated with electric scooters, it is worth noting that there has been no comparative research on injuries sustained while riding bicycles, scooters, and electric scooters within the same one-year period. Our research provides a novel comparative analysis of injury patterns from bicycles, scooters, and electric scooters over a concurrent one-year period. This study addresses a notable gap in the literature, delineating differential risk profiles and injury severities across these modalities. These findings are pivotal for informing targeted safety interventions and policymaking in urban transport safety.

In the literature, the mean age of patients with electric-scooter-related injuries has been reported to range from 32 to 36 years [3,11,12]. In our study group, the mean age of patients with e-scooter-related injuries was consistent with this trend at 31.3 years. These people were younger than the patients injured while riding a bicycle. As in most of the literature, the patients in all three modes of transport groups were predominantly male [1,4,8,11,12,17,26]. Interestingly, some mention mostly female e-scooter users [28,29]. Compared to the other groups, the scooter group had the highest proportion of female patients.

Rates of alcohol intoxication in e-scooter injuries show a wide range, varying from 4% to 50% [1,4,8,11,26,27]. Our study found that self-reported alcohol usage amongst e-scooter users was 26.8%, significantly more than in the bicycle and scooter groups.

According to our data, alcohol consumption was a significant predictor of serious and non-serious head injuries in all three vehicle groups. This has previously been demonstrated for electric scooters in a publication with fewer cases [8]; we reached a similar conclusion for larger sample sizes across all three vehicle groups. Shiffler et al. demonstrated that the neuromuscular protective reflex is crucial in protecting against head trauma [8]. When sensing instability or a sensation of fall, these reflexes cause our arms to be stretched out to protect our head, chest, and abdomen, reducing the severity of injuries and lessening the impact forces. The reflexes became delayed or suppressed in an intoxicated state, with a prolonged reaction time and worsened divided attention, auditory, and visual recognition. The standing position of e-scooter users can also explain the high frequency of head injuries among intoxicated riders, as landing on the craniomaxillofacial area is more likely due to posteroanterior movement. In another work, they also found that e-roller riders with craniomaxillofacial (CMF) trauma were 10 times more likely to be intoxicated than e-roller users without CMF trauma [8].

In our study of 1938 cases, we not only found that independent from alcohol consumption, e-scooter users were nearly 3 times more likely to suffer severe injury to the head than cyclists, but it is evident that patients, regardless of vehicle, were nearly 10 times more likely to suffer mild trauma to the head and 4 times more likely to suffer severe trauma to the head under the influence of alcohol than users not under the influence of alcohol. Furthermore, patients who did not consume alcohol sustained mild injury to the extremities nearly 4 times more frequently and severe limb and mild body injuries over 3 times more often—this may be due to using their hands to protect themselves, especially their head and core area.

As many e-scooter users rent these devices, the lack of light regulations and enforcement has led to concerns about the use of protective equipment, such as helmets, among e-scooter riders. In some studies, helmet usage was not well documented or reported as low as 1–2% [8,30,31], despite its well-understood ability to significantly reduce the risk of TBI in crashes involving skateboards, motorcycles, and bicycles [3]. Coelho et al. strongly suggest mandatory helmet usage among e-scooter riders, as they predict a 60% reduction in brain injuries due to helmet usage [17]. Mandating helmet usage is not unseen, as Denmark enacted legislation in 2022. The total effect of these laws is still unclear, but implementing mandatory helmet-use regulations can increase helmet use at specific observation sites [32].

Several studies have found that the number of injuries involving electric scooters is higher at night [1,4,28]. The same result was found by Blomberg et al., with the addition that scooters were more prevalent during the day [29]. Based on our data, comparing the three means of transportation, it is clear that most electric scooter users were injured at night, whereas patients using bicycles or regular scooters were most likely to be injured during the day. There was no relevant difference between bicycles and scooters. This difference between these three types of transportation could result from people using bikes and non-motorized scooters predominantly during the daytime for going to work and sporting purposes. In contrast, e-scooters with much higher rates of alcohol consumption are primarily used at night, suggesting a recreational purpose and an easy, cheap way of transport during the nightlife.

As we assessed the in-hospital mortality across head injury groups, we found that two patients with both severe and mild head injuries due to a bicycle accident died during the observed period. They were 81 and 82 years old at the time of the accident, and none of them wore helmets. These results correlate with the findings of the study of de Guerre et al., as they say higher age and cerebral hemorrhages were found to be independent risk factors for mortality. Also, Scott et al. stated that helmet use significantly reduces bicycle-related mortality and injury severity in bicycle-related trauma [33,34].

Across all groups, the GCS values were consistently high, reflecting predominantly mild neurological impairment among patients with head injuries. Electric scooters and scooter users exhibited nearly uniform GCS scores of 15, whereas bicycle-related cases had marginally lower averages, suggesting a greater impact force during crashes. The narrow GCS range observed (14.8–14.9) further emphasizes the importance of effective prehospital triage and early management. These findings indicate that although micromobility-related incidents frequently result in head trauma, they are typically characterized by preserved consciousness and mild injury severity.

Our study is subject to certain limitations: data were only available on those injured and who attended our hospital, not on those who did not seek medical attention. Furthermore, the number of people who were not involved in a crash while using these modes of transport is unknown, nor are the distances traveled by these vehicles. As a result, it is impossible to deduce from patient volumes which mode of transport presents a greater risk, and observations could be made only regarding injury patterns and circumstances. There was also no record of whether patients used their own e-scooter or a rented device. Recording the use of helmets and other protective equipment would have been valuable, but it was impossible due to documentation inconsistencies. Most data on alcohol consumption were based on self-report, which is prone to social desirability and recall bias, potentially leading to underestimation of alcohol involvement. Although a considerable proportion of patients reported intoxication, caution is warranted when interpreting its impact on injury severity, as the actual effect might be larger than observed.

Initially, our study characterized injuries by body region and severity (mild or severe), which, while systematic, did not fully align with standardized measures to facilitate comparative analysis across studies. Subsequent studies may be necessary with more standardized data, such as the Injury Severity Score (ISS) or the need for surgical intervention, to facilitate better consistency and comparability across studies.

According to our research findings, it is evident that alcohol impairment poses a substantial risk of head injuries. Our research suggests that people under the influence of alcohol are at greater risk of severe craniofacial injury than sober people. However, electric scooters also increase the risk of serious head injury compared to cycling and manual scooters. McGuinness et al., in a comparative work between e-scooter users and cyclists, found a significantly higher BAC in e-scooter users and a tendency to injure more frequently at night than cyclists, which coincides with our findings. They suggest that e-scooters may not be as safe as cycling [35].

Therefore, considering the significantly higher incidence of severe head injuries observed in e-scooter users, with the higher incidence of alcohol consumption in this group, routine cranial CT scans for patients who consumed alcohol, particularly those receiving trauma care following an e-scooter crash, are worth considering. Our data, which includes an exceptionally high rate of alcohol consumption among e-scooter users and predominantly night-time injuries, suggests that this mode of transportation is primarily used for recreational purposes and as easy transportation during nightlife. This fact, combined with the large number of e-scooter-related serious injuries, raises the need for a review of legal regulations, stricter monitoring of alcohol use, and more stringent measures in enforcing protective gear.

## 5. Conclusions

Our findings indicate that patients who consumed alcohol and patients injured using electric scooters are more prone to severe craniofacial injuries than completely sober riders, cyclists, and scooter users. To prevent such severe injuries, it is necessary to revise the legal framework by making helmets compulsory and implementing stricter regulations and controls for alcohol consumption before using an e-scooter. This is particularly important considering the prevalent use of e-scooters for recreation and night-time transportation.

The data shows that e-scooter riders are at a higher risk of injury during night-time hours, have a higher incidence of alcohol consumption, and are more likely to sustain head injuries compared to cyclists. Furthermore, over half of the injuries that occurred at night were sustained by e-scooter riders. These findings highlight the importance of helmet use in preventing head injuries among e-scooter riders.

## Figures and Tables

**Table 1:** Descriptive statistics of the study population, SD: Standard deviation, *: Corrected for unequal category intervals; rounded values are shown.

	Bike (N = 1378)	Scooter (N = 190)	E-Scooter (N = 370)	Overall (N = 1938)
Age (years)				
Mean (SD *)	38.5 (14.9)	31.2 (13.7)	31.9 (10.9)	36.5 (14.4)
Sex—male	954 (69.2%)	112 (58.9%)	245 (66.2%)	1311 (67.6%)
Daytime—corrected *				
Morning	114 (8.3%)	17 (9.1%)	25 (6.8%)	157 (8.1%)
Daytime	596 (43.3%)	80 (41.9%)	79 (21.2%)	754 (38.9%)
Evening	540 (39.2%)	67 (35.0%)	97 (26.3%)	704 (36.3%)
Night	127 (9.2%)	27 (14.0%)	169 (45.6%)	322 (16.6%)
Alcohol consumption	63 (4.6%)	11 (5.8%)	99 (26.8%)	173 (8.9%)
Hospital admissions	142 (10.3%)	13 (6.8%)	54 (14.6%)	209 (10.8%)
Mean duration of stay in hospital (days) (SD)	2.30	1.83	3.13	

**Table 2:** Injury types by vehicle and alcohol intoxication.

	Bike		Scooter		E-Scooter	
	Sober (N = 1315)	Consumed Alcohol (N = 63)	Sober (N = 179)	Consumed Alcohol (N = 11)	Sober (N = 271)	Consumed Alcohol (N = 99)
Mild limb	1058 (80.5%)	29 (46.0%)	137 (76.5%)	5 (45.5%)	214 (79.0%)	48 (48.5%)
Severe limb	342 (26.0%)	6 (9.5%)	44 (24.6%)	1 (9.1%)	81 (29.9%)	13 (13.1%)
Mild head	361 (27.5%)	51 (81.0%)	61 (34.1%)	9 (81.8%)	99 (36.5%)	89 (89.9%)
Severe head	37 (2.8%)	8 (12.7%)	7 (3.9%)	4 (36.4%)	14 (5.2%)	20 (20.2%)
Spinal injury	57 (4.3%)	2 (3.2%)	2 (1.1%)	0 (0%)	5 (2.6%)	0 (0%)
Mild body	180 (13.7%)	4 (6.3%)	12 (6.7%)	1 (9.1%)	31 (11.4%)	3 (3.0%)
Severe body	29 (2.2%)	0 (0%)	1 (0.6%)	0 (0%)	6 (2.2%)	1 (1.0%)

**Table 3:** In-hospital mortality, surgery rates, and GCS distributions across head injury groups.

	*Type*	*N*	*GCS 15, n (%)*	*GCS 14, n (%)*	*GCS 13, n (%)*	*GCS 12, n (%)*	*GCS 10, n (%)*	*GCS 9, n (%)*	*Unknown GCS*	*In-Hospital Death, n (%)*	*Mean GCS ± SD*	*Surgery, n (%)*
*Electric-scooter-related head injury*	Severe	34	31 (91.17)	2 (5.88)	0(0.00)	1 (2.94)	0(0.00)	0(0.00)	0(0.00)	0 (0.00)	14.85 ± 0.56	2 (5.88)
	Mild	188	182 (96.81)	2 (1.06)	1 (0.53)	1 (0.53)	1 (0.53)	0(0.00)	1 (0.53)	0 (0.00)	14.94 ± 0.46	9 (4.79)
*Bicycle-related head injury*	Severe	45	43 (95.56)	0(0.00)	0(0.00)	1 (2.22)	0(0.00)	1 (2.22)	0(0.00)	2 (4.44)	14.80 ± 0.99	6 (13.33)
	Mild	412	407 (98.79)	2 (0.46)	1 (0.24)	1 (0.24)	0(0.00)	1 (0.24)	0(0.00)	2 (0.48)	14.97 ± 0.35	15 (3.64)
*Scooter-related head injury*	Severe	11	10 (90.91)	1 (9.09)	0(0.00)	0(0.00)	0(0.00)	0(0.00)	0(0.00)	0 (0.00)	14.91 ± 0.30	0 (0.00)
	Mild	70	67 (95.71)	2 (2.86)	0(0.00)	1 (1.43)	0(0.00)	0(0.00)	0(0.00)	0 (0.00)	14.93 ± 0.39	0 (0.00)

## Data Availability

The original data can be available upon request.
